# Ganglioneuroma: a rare appendiceal tumour – case report and literature review

**DOI:** 10.1093/jscr/rjae735

**Published:** 2024-12-16

**Authors:** Rakesh Quinn, Jodie Ellis-Clark

**Affiliations:** Department of Colorectal Surgery, Nepean Hospital, Derby St, Kingswood, NSW, 2747, Australia; University of Sydney, Sydney Medical School, Faculty of Medicine and Health, NSW, Australia; Department of Colorectal Surgery, Nepean Hospital, Derby St, Kingswood, NSW, 2747, Australia

**Keywords:** ganglioneuroma, appendix, colonoscopy, colorectal neoplasms

## Abstract

Ganglioneuromas (GN) are tumours of ectodermal origin, derived from the neural crest cells. Appendiceal GN are extremely rare, with only eight contemporary case reports in the literature. Being benign and indolent, the necessity of resection for GNs is often debated. However, obtaining tissue to confirm the diagnosis can be challenging, frequently leading to surgical resection. We present a case of an 85-year-old male with an enlarging appendiceal nodule diagnosed endoscopically. Further investigation with computed tomography (CT) scan failed to define the pathology. A laparoscopic appendicectomy was performed, which confirmed the diagnosis of appendiceal GN.

## Introduction

Neoplastic disease of the appendix is found in 0.7 to 1.7% of appendicectomies, with carcinoid and epithelial tumours being the most common pathology [1]. Ganglioneuromas (GN) are derived from neural crest cells associated with the autonomic ganglia, are an exceedingly rare finding in the appendix [2]. GNs typically present within the first to fifth decades of life, with a 1.6:1 female predominance [3]. Majority of GNs (97.6%) present sporadically, with the remainder associated with genetic syndromes such as neurofibromatosis type 1 (NF1) and type 2 (NF2), and multiple endocrine neoplasia type 2 (MEN 2) [4, 5]. GNs are classified into three groups: polypoid GN, ganglioneuromatous polyposis or diffuse ganglioneuromatosis [4]. Solitary polypoid GNs are rarely associated with genetic syndromes.

GNs are considered a benign lesion with an indolent course and a very low potential for malignant transformation [4, 6, 7]. They are largely asymptomatic, though intestinal manifestations, such as bleeding, abdominal pain, obstruction, anaemia, appendicitis, and perforation have been described. Histopathological diagnosis with biopsy can be unreliable, and there are no distinct characteristics identifiable on imaging modalities [3]. With no clear guidelines on whether surgical intervention is required for all GN, the difficulty of pre-operative diagnosis often leads to surgical resection.

## Case report

**Figure 1:**
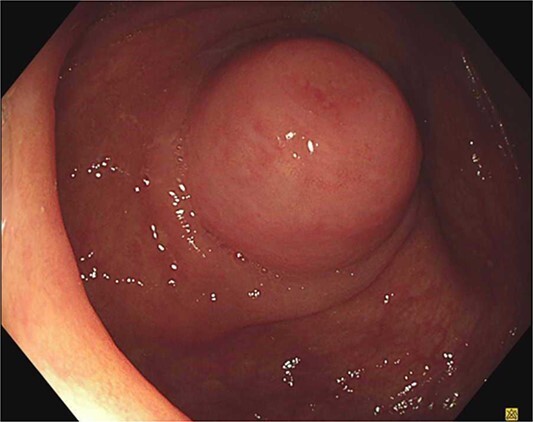
Colonoscopic view of the appendiceal orifice.

An 85-year-old male was referred to a colorectal surgeon with an appendiceal nodule bulging into the caecum on routine colonoscopy (Fig. 1). A considerably smaller protuberance was noted 5 years prior with a plan for surveillance, he was asymptomatic to the appendiceal nodule and otherwise only reported dyspeptic symptoms. He has a background of peptic ulcers, obstructive sleep apnoea, hypertension and laparoscopic cholecystectomy. He has no known history of genetic syndromes. A CT abdomen-pelvis was organized to further define the appendiceal pathology. The appendix was of normal calibre, without definite nodular thickening and comparable to a previous study in 2019. Further there was no evidence of lymphadenopathy or metastatic disease.

**Figure 2:**
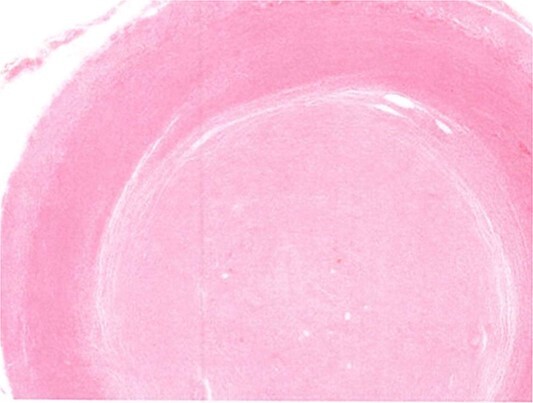
Appendiceal lumen obliterated by neural tumour.

**Figure 3:**
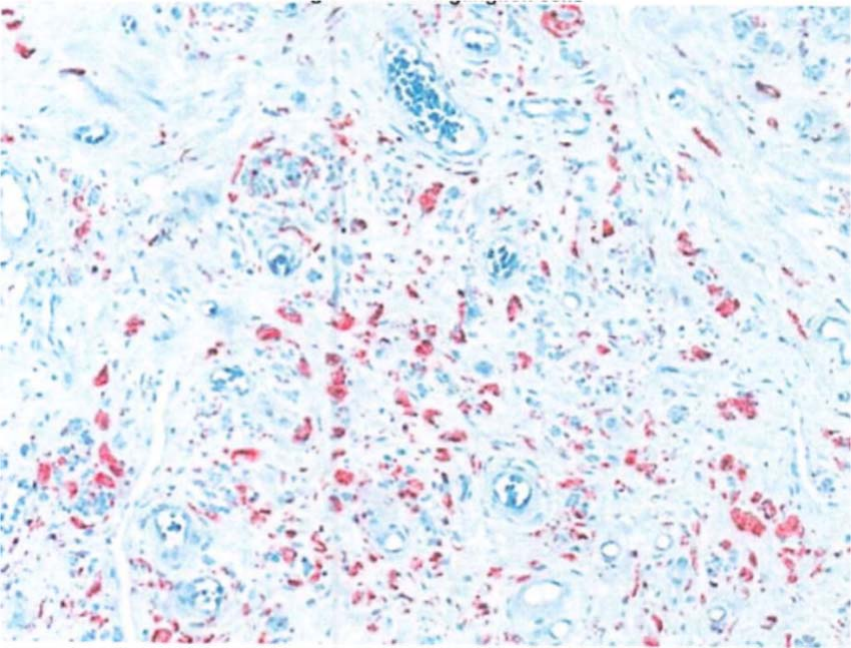
S100 neural marker positive.

Due to the interval increase in size on colonoscopy, the patient proceeded to a laparoscopic stapled caecotomy, for which he recovered well and was discharged Day 1 post-operatively. The histopathology report found a benign neural tumour bulging into the appendiceal orifice and obliterating the lumen (Fig. 2). The tumour was located in the base of the appendix, 15 mm from the resection margin. It contained Schwann cells admixed with mast cells and ganglion cells which stained positive for S100 (Fig. 3). The findings were consistent with a benign mature GN.

## Discussion

GNs, being of ectodermal origin, can arise from nearly every organ within the body. However, reports of appendiceal GNs are exceedingly rare. The most extensive description is provided by Collins *et al.* [2] in 1963, who reviewed 71 000 appendicectomy specimens and found that 0.28% contained a GN. A review of the literature identified only nine contemporary case reports, including the current case (see Table 1). Over half of the cases were associated with a genetic syndrome, most commonly NF1. Three patients, including our case, were asymptomatic, with the most common symptomatic presentation being acute appendicitis. Appendicectomy was described as the surgical procedure of choice, with only two studies performing a hemicolectomy for diffuse ganglioneuromatosis associated with NF1.

**Table 1:** Appendiceal ganglioneuroma case reports

	**Author (Year)**	**Age**	**Gender**	**Presentation**	**Genetic syndrome**	**Procedure**	**Histopathology**	**Size**	**Location**
1	Zarabi *et al.* (1982) [8]	27	M	Appendicitis		Appendicectomy	Ganglioneuroma	2 cm	Mid portion
2	Lie *et al.* (1992) [9]	15	M	Abdominal mass	NF1	Right hemicolectomy	Diffuse ganglioneuromatosis		
3	Lockhart *et al.* (2000) [10]	33	F	Pain (6mths), haematochezia	NF1	Partial right colectomy	Diffuse ganglioneuromatosis		
4	Gonzalez *et al.* (2016) [5]	13	F	Appendicitis	PTEN	Appendicectomy	Ganglioneuroma	1 × 0.4 cm	Tip
5	Esteron *et al.* (2017) [11]	30	F	Dysparenuria, urinary frequency	NF2	Appendicectomy	Ganglioneuroma	6 × 3.5 cm	Distal
6	Shimizu *et al.* (2021) [12]	29	M	Asymptomatic, NF1 surveillance CT	NF1	Appendicectomy	Ganglioneuroma	5 × 3.5 cm	
7	Koullouros *et al.* (2022) [13]	30	M	Appendicitis		Appendicectomy	Ganglioneuroma		Proximal
8	Pachon *et al.* (2024) [14]	42	M	Asymptomatic, exenteration for rectal cancer		Exenteration, prophylactic appendicectomy	Ganglioneuroma		Tip
9	*Current case*	85	M	Asymptomatic, colonoscopy finding		Caecetomy	Ganglioneuroma		Proximal

Pre-operative diagnosis of appendiceal GN can be a challenge, as appendiceal tumours are rarely exposed through the appendiceal orifice, making it difficult to obtain sufficient tissue for diagnosis via colonoscopy [12]. Additionally, GNs can be hormonally active, with 16.7% cases reported in the literature associated with elevated catecholamines or vasoactive intestinal peptide, posing a risk of hypertensive crisis following biopsy [3]. Pre-operative imaging with CT and magnetic resonance imaging (MRI) have been described but is non-specific with only 24.1% suspected as GN on imaging [3, 4]. Fludeoxyglucose-positron emission tomography (FDG-PET) has been performed in a small subset of patients and found 73.7% of GN showed avidity [3]. Thus, consistent with all nine case reports, the diagnosis of appendiceal GN remains primarily a pathological one following surgical resection.

Management strategies for appendiceal GN, which are often asymptomatic benign tumours, are informed by principles applied to other benign tumours within the appendix, with surgical resection via simple appendicectomy deemed sufficient [4, 5]. However, post-resection surgical morbidity has led to two studies challenging the necessity of resection. Retrosi *et al.* [6] reported on 23 patients undergoing resection for GN, with a 30% complication rate, including Horner’s syndrome, chylothorax, and bowel obstruction. Importantly, 43% had involved margins with no tumour progression or recurrence noted at follow-up of 33.5 months. Similarly, Sanchez-Galan *et al.* [7], reporting on 24 patients with a complication rate of 25%, and incomplete resection in 16% with no regrowth or malignant behaviour noted at a follow-up of 84 months. Further the Transatlantic Australasian Retroperitoneal Sarcoma Working Group reviewed 328 patients with retroperitoneal-abdominal-pelvic GNs, they found of the 35.4% who underwent active surveillance, only 5.8% demonstrated tumour growth [4]. They therefore recommended, non-operative management with serial imaging may be appropriate in biopsy proven, asymptomatic GNs. In our case, the patient did not report any symptoms from the GN, despite a significant increase in size noted endoscopically. But due to our lack of tissue diagnosis and given appendicitis is the most common symptomatic presentation of appendiceal tumours, we recommended resection to exclude malignancy but also to pre-empt the eventual acute presentation [1, 15]. Emergency surgery in an 85-year-old carries greater morbidity risk than an elective laparoscopic appendicectomy, guiding our management.

## Conclusion

GNs are an extremely rare entity within the appendix. Despite their benign and indolent nature, surgical resection remains the mainstay treatment especially when there is diagnostic uncertainty. The ultimate decision between surgical management and surveillance should be individualized, considering tumour characteristics, symptoms and surgical risk.
